# Supplementary material for: Reannotation of the Ribonucleotide Reductase in a Cyanophage Reveals Life History Strategies Within the Virioplankton
Source: Front Microbiol. 2019 Feb 5;10:134. doi: 10.3389/fmicb.2019.00134 (PMC6370689; doi:10.3389/fmicb.2019.00134)
Supplement: Supplementary file 1 [file Data_Sheet_1.PDF]

Supplementary Material

Reannotation of the ribonucleotide reductase in a cyanophage reveals life history strategies within the virioplankton

Amelia O Harrison, Ryan M Moore, Shawn W Polson, K Eric Wommack*

*** Correspondence:** Corresponding Author: wommack@dbi.udel.edu

1 Supplementary Methods

Protein sequence similarity networks (SSN) were constructed with the same trimmed RNR sequences used for the Class I α with Class II phylogeny and for the Class I α-only phylogeny. Cyano SP α subunit sequences trimmed to the corresponding region were also included. The SSNs were generated with the Enzyme Similarity Tool (EFI-EST) (Gerlt et al., 2015) as in Rose et al. (Evalue: 5, fraction: 1) (Rose et al., 2018), except that multiple alignment scores (25 and 40 for the Class I α with Class II sequences, 110 and 155 for Class I α-only sequences) were chosen to generate a more complete picture of protein connectivity. As the full networks were too large to visualize in Cytoscape (Shannon et al., 2003; Smoot et al., 2011) representative node networks were presented instead (for Class I α with Class II sequences: 55% clustering for alignment score 25, 60% clustering for alignment score 40; for Class I α-only sequences: 40% clustering for alignment score 110, 55% for alignment score 155).

2 Supplementary Figures and Tables

2.1 Supplementary Tables

Supplementary Table 1. Accession numbers for annotated Class I α, Class I β, and Class II references used for alignment of putative α and candidate β subunits and curation of phylogenetic reference sequences.

Class I α subunit representatives

Class	Subtype/ Subclass	Species name	Crystal structure?	Crystal structure PDB* id	Reference	Accession no.
I	a	*Escherichia coli* str. K-12	yes	1RLR	(Uhlin and Eklund, 1994)	CQR81730.1
	b	*Salmonella typhimurium (strain LT2 / SGSC1412 / ATCC 700720)*	yes	1PEU	(Uppsten et al., 2003)	WP_000246626.1
	c	*Chlamydia trachomatis* str. D/UW-3/Cx	no	N/A	(Högbom et al., 2004)	WP_009872213.1
	d	*Flavobacterium johnsoniae*	no	N/A	(Rose et al., 2018)	WP_012026039.1
	e	*Aerococcus urinae*	no	N/A	(Blaesi et al., 2018)	WP_060778521.1

Class I β subunit representatives

Class	Subclass	Species name	Crystal structure?	Crystal structure PDB id*	Reference	Accession no.
I	a	*Escherichia coli* str. K-12	yes	1RIB	(Nordlund and Eklund, 1993)	KXG99827.1
	b	*Salmonella typhimurium (strain LT2 / SGSC1412 / ATCC 700720)*	yes	1R2F	(Eriksson et al., 1998)	WP_000777903.1
	c	*Chlamydia trachomatis* str. D/UW-3/Cx	yes	1SYY; 4D8F	(Högbom et al., 2004); (Dassama et al., 2012)	WP_009872214.1
	d	*Flavobacterium johnsoniae*	yes	6CWO-P	(Rose et al., 2018)	WP_012026040.1
	e	*Aerococcus urinae*	yes	6EBO	(Blaesi et al., 2018)	WP_013669290.1

Class II Representatives

Class	Subclass	Species name	Crystal structure?	Crystal structure PDB id*	Reference	Accession no.

II	monomeric, RTPR	*Lactobacillus leichmanii*	yes	1L1L	(Sintchak et al., 2002)	AAA03078.1
	dimeric	*Thermotoga maritima*	yes	3O0N	(Larsson et al., 2004)	WP_004082700.1

* PDB = Protein Data Bank

Supplementary Table 2. Catalytic residues in Class I RNR α and β subunits and their positions in the putative α and β sequences from *Prochlorococcus* phage P-SSP7. Residues in **bold** were used in reference curation.

RNR α subunit

Residue	Position in P-SSP7	Position in *E. coli*	Function	Citation
**C**	32	225	active site disulfide bridge	(Lin et al., 1987); (Booker et al., 1994)
Q	105R	294	substrate specificity	(Ahmad et al., 2012)
R	110	298	substrate specificity	(Ahmad et al., 2012)
N	187	437	hydrogen bonds	(Kasrayan et al., 2002)
**C**	189	439	thiyl radical - abstracts H	(Mao et al., 1992a); (Mao et al., 1992b)
**E**	191	441	hydrogen bonds	(Persson et al., 1997)
**C**	200	462	active site disulfide bridge	(Mao et al., 1992a)
**Y**	423	730	radical transfer	(Uhlin and Eklund, 1994)
**Y**	424	731	radical transfer	(Uhlin and Eklund, 1994)
C	464	754	radical transfer	(Lin et al., 1987); (Booker et al., 1994)
C	466	759	radical transfer	(Lin et al., 1987); (Booker et al., 1994)

RNR β subunit

Residue	Position in P-SSP7	Position in *E. coli*	Function	Citation
**W**	14	48	reduces dioxygen	(Baldwin et al., 2000); (Krebs et al., 2000)
**Y**	78L	122	protein radical	(Larsson and Sjöberg, 1986)
F	122	208	protein radical stability	(Ormö Mats, 1995)
F	127	212	protein radical stability	(Ormö Mats, 1995)
R	145	236	radical transport	(Nordlund and Eklund, 1993); (Eklund et al., 2001)
**Y**	236	356	radical transport	(Climent et al., 1992); (Rova et al., 1999)

Supplementary Table 3. Clustering statistics for Class I α subunit sequences removed from RNRdb groups NrdE and NrdAz prior to phylogenetic analysis.

Group NrdAz			
	No. clusters (% of total sequences)		
**Cluster identity**	Only included sequences	Mixed	Only excluded sequences
70	23 (0.7)	46 (98.2)	9 (1.2)
75	43 (1.9)	89 (96.8)	17 (1.4)
80	94 (3.4)	164 (95.1)	24 (1.5)

Group NrdE			
	No. clusters (% of total sequences)		
**Cluster identity**	Only included sequences	Mixed	Only excluded sequences
70	27 (1.7)	40 (98.2)	7 (0.2)
75	60 (2.0)	64 (97.7)	11 (0.2)
80	114 (4.2)	103 (95.5)	14 (0.3)

Supplementary Table 4. Accession numbers for genomes and RNR and Pol I protein sequences of the Cyano SP clade.

**Virus**	**Genome accession no.**	**RNR α accession no.**	**RNR β accession no.**	**Pol I accession no.**
Prochlorococcus phage P-SSP7	NC_006882.2	YP_214197.1	YP_214198.1	YP_006355438.1
Cyanophage P-SSP2	NC_016656.1	YP_005087372.1	YP_005087373.1	frameshift
Cyanophage 9515-10a	NC_016657.1	YP_005087443.1	YP_005087442.1	frameshift
Cyanophage NATL1A-7	NC_016658.1	YP_005087467.1	YP_005087466.1	frameshift
Cyanophage NATL2A-133	NC_016659.1	YP_005087553.1	YP_005087552.1	frameshift
Cyanophage SS120-1	NC_020872.1	YP_007676889.1	YP_007676888.1	YP_007676894.1
Cyanophage Syn5	NC_009531.1	YP_001285442.1	YP_001285443.1	YP_001285436.1
Synechococcus phage S-CBS4	NC_016766.1	YP_005098300.1	YP_005098301.1	frameshift

2.2 Supplementary Figures

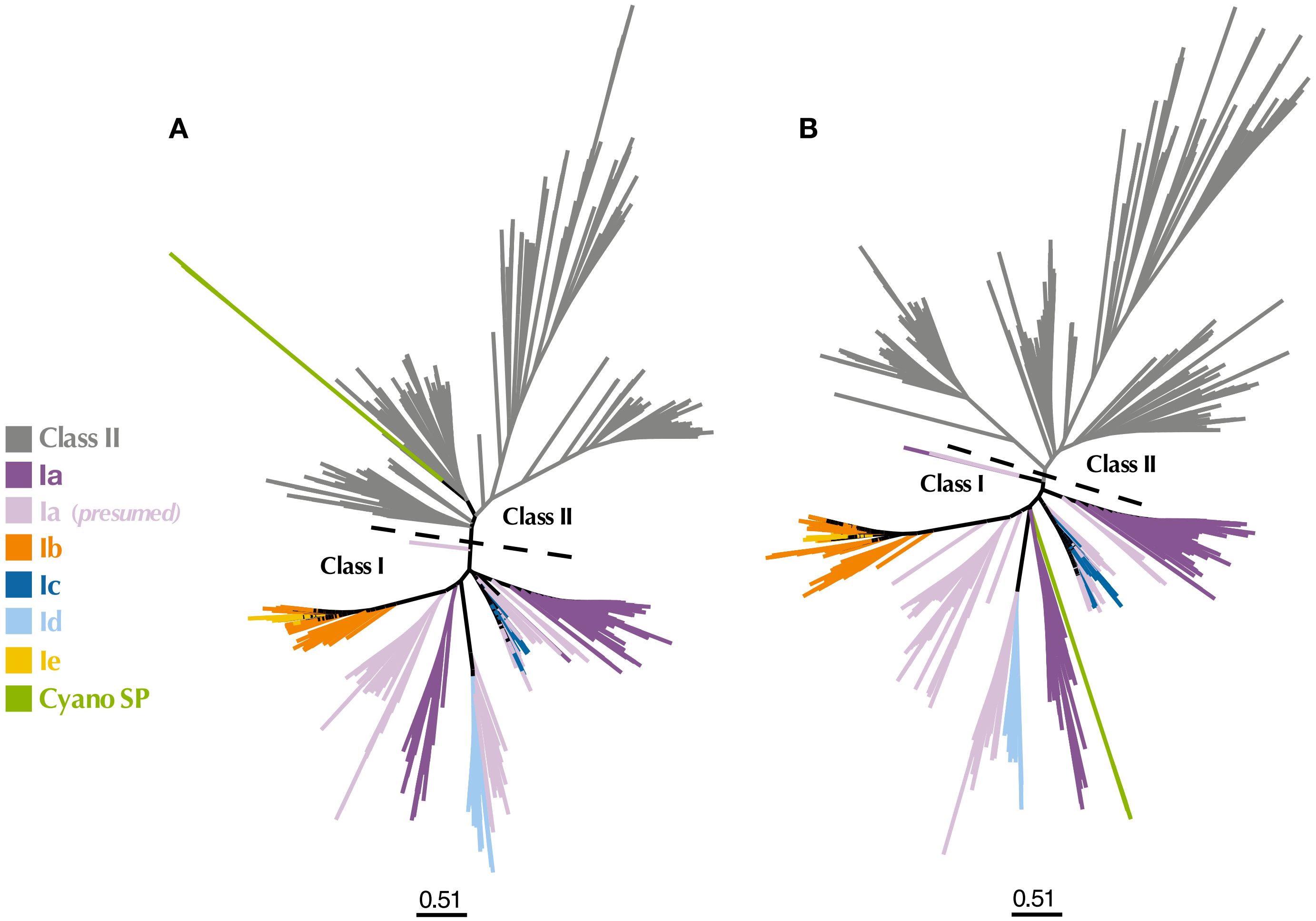

Supplementary Figure 1. Maximum-likelihood phylogenetic tree of trimmed Cyano SP clade α subunits with Class I α and Class II sequences from the RNRdb trimmed to a region of interest and clustered at **A)** 70% and **B)** 75%. Gray branches belong to Class II. Colored branches belong to one of the five Class I subclasses, or Cyano SP as indicated in the key. Light purple branches indicate RNRdb clades without characterized members, which are assumed to be subclass Ia enzymes. Trees were constructed using FastTree (Price et al., 2010) and visualized and customized in Iroki (Moore et al., 2018). Scale bars represent amino acid changes per 100 positions.

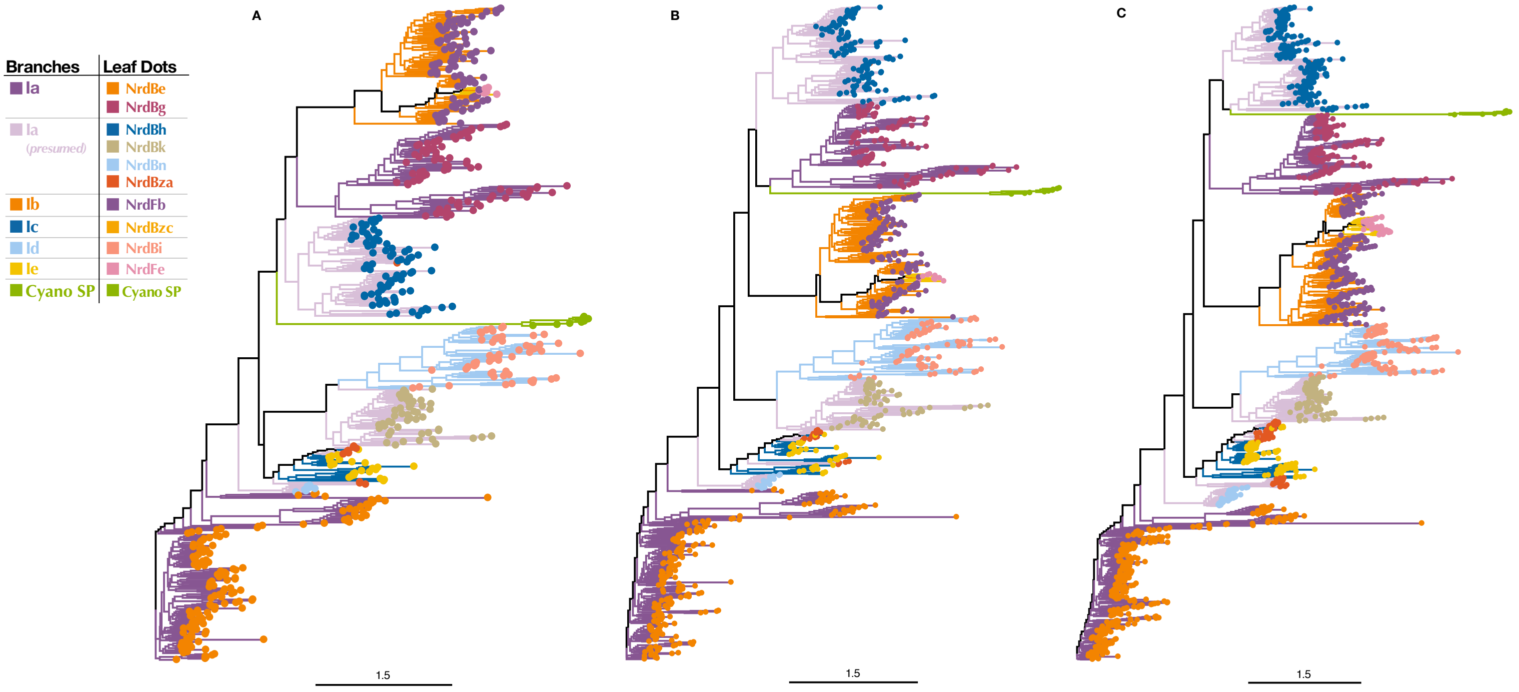

Supplementary Figure 2. Maximum-likelihood phylogenetic trees of trimmed Cyano SP β subunits with trimmed RNRdb Class I β subunits clustered at **A)** 70%, **B)** 75%, and **C)** 80%. Branches are colored by Class I subclass. Leaf dots are colored by RNRdb group. Both branches and leaf dots indicating the Cyano SP clade are colored light green. Scale bars represent amino acid changes per 100 positions.

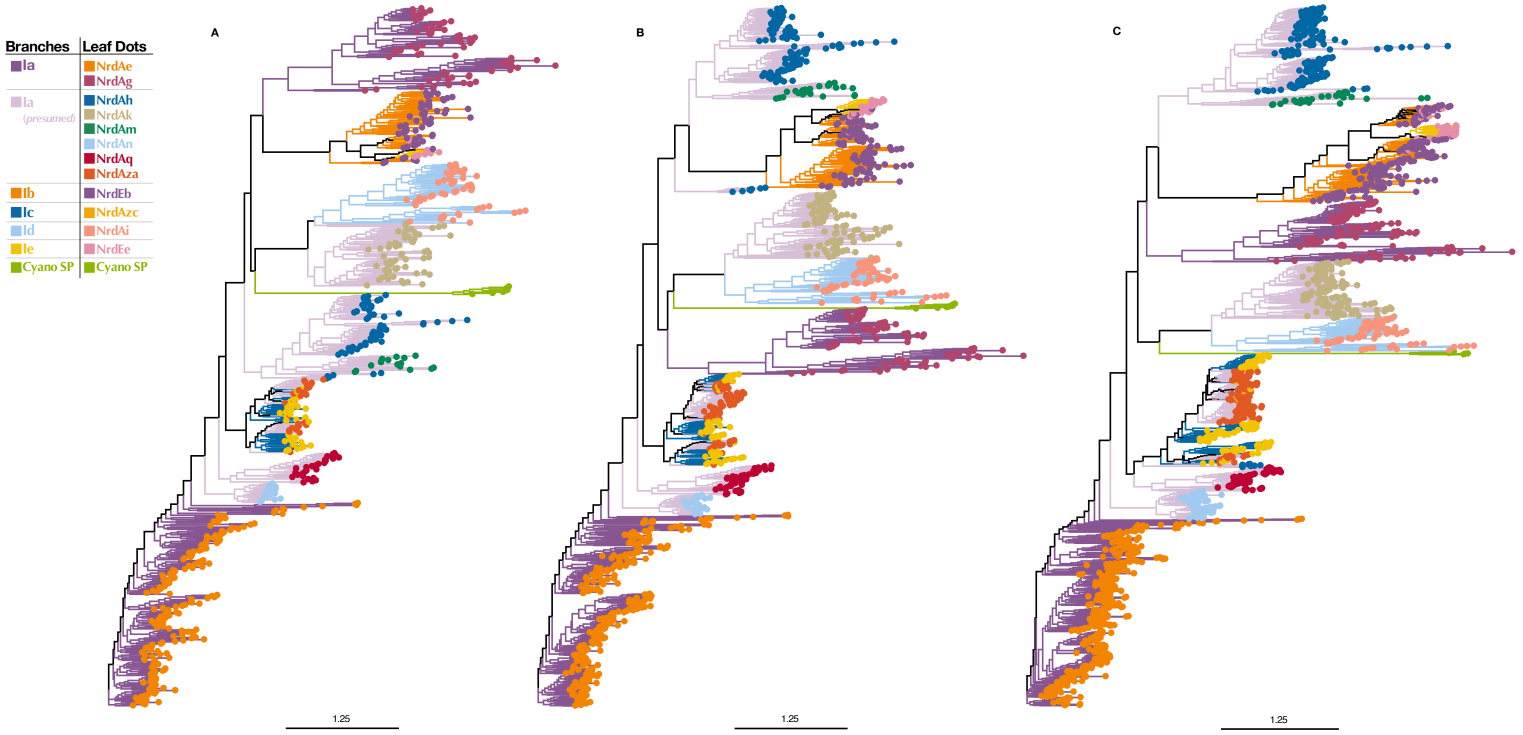

Supplementary Figure 3. Maximum-likelihood phylogenetic trees of trimmed Cyano SP α subunits with trimmed RNRdb Class I α subunits clustered at **A)** 70%, **B)** 75%, and **C)** 80%. Branches are colored by Class I subclass. Leaf dots are colored by RNRdb group. Both branches and leaf dots indicating the Cyano SP clade are colored light green. Colors correspond to figures in the main body of the paper. Scale bars represent amino acid changes per 100 positions.

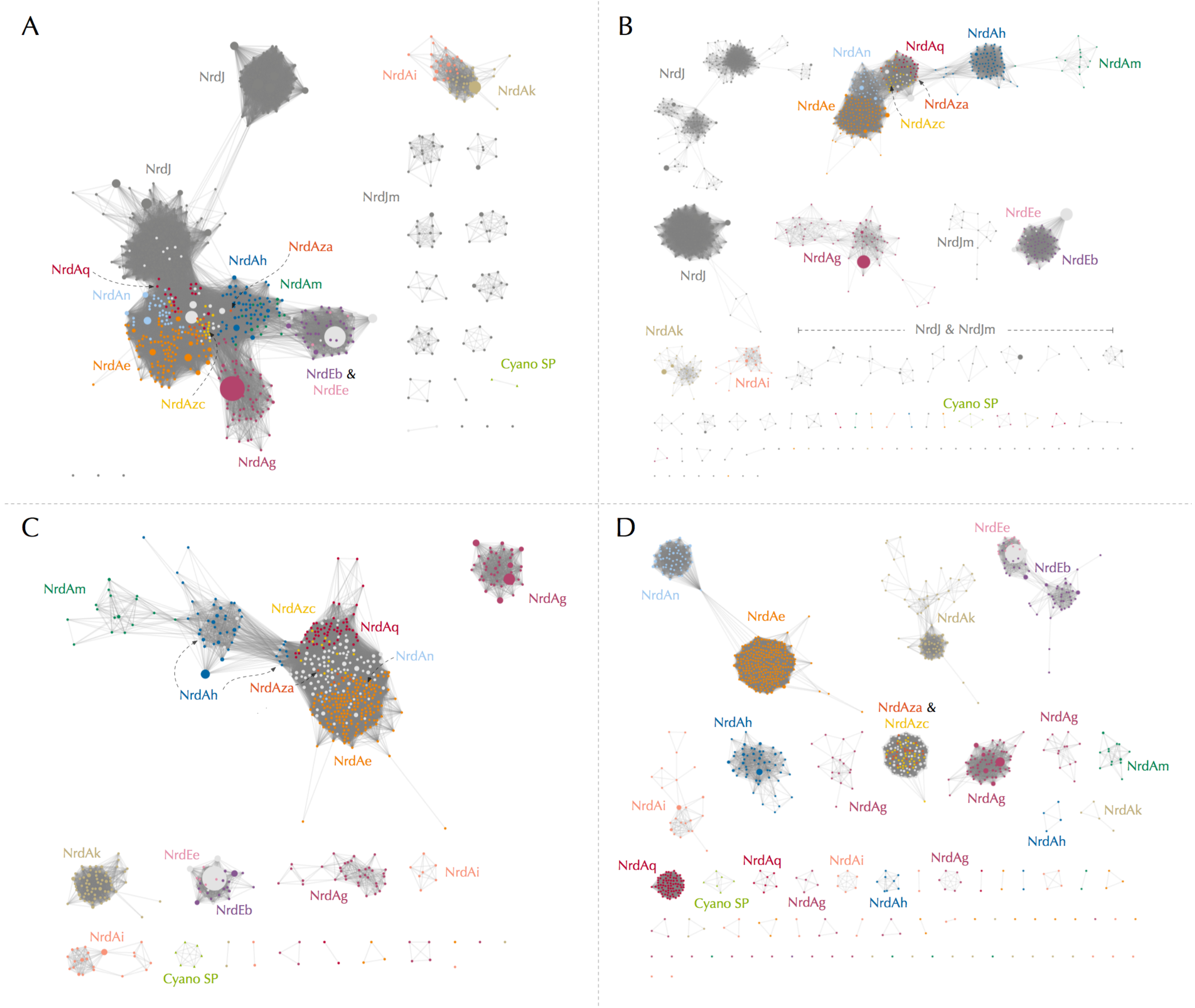

Supplementary Figure 4. Protein SSNs for Class I α with Class II sequences (**A** and **B**) and for Class I α-only sequences (**C** and **D**) used for phylogenetic analyses. Nodes are colored based on RNRdb group and match leaf dot colors on the trees in Fig. S3. In **A** and **B**, nodes representing Class II sequences are colored dark grey. Nodes labeled NrdJm are monomeric Class II and nodes labeled NrdJ are dimeric Class II. In all networks, light grey nodes contain sequences from more than one RNRdb group. Larger nodes represent larger clusters of sequences. In **A)** nodes represent sequence clusters ≥ 55% similarity and edges connect nodes with alignment scores ≥ 25. In **B)** nodes represent sequence clusters ≥ 60% similarity and edges connect nodes with alignment scores ≥ 40. In **C)** nodes represent sequence clusters ≥ 40% similarity and edges connect nodes with alignment scores ≥ 110. In **D)** nodes represent sequence clusters ≥ 55% similarity and edges connect nodes with alignment scores ≥ 155.

3 Supplementary References

Ahmad, M. F., Singh Kaushal, P., Wan, Q., Wijerathna, S. R., An, X., Huang, M., et al. (2012). Role of Arginine 293 and Glutamine 288 in Communication between Catalytic and Allosteric Sites in Yeast Ribonucleotide Reductase. *J. Mol. Biol.* 419, 315–329. doi:10.1016/j.jmb.2012.03.014.

Baldwin, J., Krebs, C., Ley, B. A., Edmondson, D. E., Huynh, B. H., and Bollinger Jr., J. M. (2000). Mechanism of Rapid Electron Transfer during Oxygen Activation in the R2 Subunit of Escherichia coli Ribonucleotide Reductase. 1. Evidence for a Transient Tryptophan Radical. *J. Am. Chem. Soc.* 122, 12195–12206. doi:10.1021/JA001278U.

Blaesi, E. J., Palowitch, G. M., Hu, K., Kim, A. J., Rose, H. R., Alapati, R., et al. (2018). Metal-free class Ie ribonucleotide reductase from pathogens initiates catalysis with a tyrosine-derived dihydroxyphenylalanine radical. *Proc. Natl. Acad. Sci. U. S. A.*, 201811993. doi:10.1073/pnas.1811993115.

Booker, S., Licht, S., Broderick, J., and Stubbe, J. (1994). Coenzyme B 12-Dependent Ribonucleotide Reductase: Evidence for the Participation of Five Cysteine Residues in Ribonucleotide Reductiont. *Biochemistry* 33, 12676–12685.

Climent, I., Sjöberg, B. M., and Huang, C. Y. (1992). Site-directed mutagenesis and deletion of the carboxyl terminus of *Escherichia coli* ribonucleotide reductase protein R2. Effects on catalytic activity and subunit interaction. *Biochemistry* 31, 4801–4807. doi:10.1021/bi00135a009.

Dassama, L. M. K., Boal, A. K., Krebs, C., Rosenzweig, A. C., Bollinger, J. M., and Jr. (2012). Evidence that the β subunit of *Chlamydia trachomatis* ribonucleotide reductase is active with the manganese ion of its manganese(IV)/iron(III) cofactor in site 1. *J. Am. Chem. Soc.* 134, 2520–2523. doi:10.1021/ja211314p.

Eklund, H., Uhlin, U., Färnegårdh, M., Logan, D. T., and Nordlund, P. (2001). Structure and function of the radical enzyme ribonucleotide reductase. *Prog. Biophys. Mol. Biol.* 77, 177–268. doi:10.1016/S0079-6107(01)00014-1.

Eriksson, M., Jordan, A., and Eklund, H. (1998). Structure of Salmonella typhimurium nrdF Ribonucleotide Reductase in Its Oxidized and Reduced Forms. *Biochemistry* 37, 13359–13369.

Gerlt, J. A., Bouvier, J. T., Davidson, D. B., Imker, H. J., Sadkhin, B., Slater, D. R., et al. (2015). Enzyme Function Initiative-Enzyme Similarity Tool (EFI-EST): A web tool for generating protein sequence similarity networks. *Biochim. Biophys. Acta* 1854, 1019–1037. doi:10.1016/j.bbapap.2015.04.015.

Högbom, M., Stenmark, P., Voevodskaya, N., McClarty, G., Gräslund, A., and Nordlund, P. (2004). The Radical Site in Chlamydial Ribonucleotide Reductase Defines a New R2 Subclass. *Science.* 305, 245–248.

Kasrayan, A., Persson, A. L., Sahlin, M., and Sjöberg, B. M. (2002). The conserved active site asparagine in class I ribonucleotide reductase is essential for catalysis. *J. Biol. Chem.* 277, 5749–5755. doi:10.1074/jbc.M106538200.

Krebs, C., Chen, S., Baldwin, J., Ley, B. A., Patel, U., Edmondson, D. E., et al. (2000). Mechanism of Rapid Electron Transfer during Oxygen Activation in the R2 Subunit of Escherichia coli Ribonucleotide Reductase. 2. Evidence for and Consequences of Blocked Electron Transfer in the W48F Variant. *J. Am. Chem. Soc.* 122, 12207–12219. doi:10.1021/JA001279M.

Larsson, A., and Sjöberg, B. M. (1986). Identification of the stable free radical tyrosine residue in ribonucleotide reductase. *EMBO J.* 5, 2037–2040. doi:10.1002/J.1460-2075.1986.TB04461.X.

Larsson, K.-M., Jordan, A., Eliasson, R., Reichard, P., Logan, D. T., and Nordlund, P. (2004). Structural mechanism of allosteric substrate specificity regulation in a ribonucleotide reductase. *Nat. Struct. Mol. Biol.* 11, 1142–9. doi:10.1038/nsmb838.

Lin, A.-N. I., Ashley, G. W., and Stubbe, J. A. (1987). Location of the Redox-Active Thiols of Ribonucleotide Reductase: Sequence Similarity between the *Escherichia coli* and *Lactobacillus leichmannii* Enzymes. *Biochemistry* 26, 6905–6909.

Mao, S. S., Holler, T. P., Yu, G. X., Bollinger, J. M., Booker, S., Johnston, M. I., et al. (1992a). A Model for the Role of Multiple Cysteine Residues Involved in Ribonucleotide Reduction: Amazing and Still Confusing. *Biochemistry*. doi:10.1021/bi00155a029.

Mao, S. S., Yu, G. X., Chalfoun, D., and Stubbe, J. (1992b). Characterization of C439SR1, a Mutant of *Escherichia coli* Ribonucleotide Diphosphate Reductase: Evidence That C439 Is a Residue Essential for Nucleotide Reduction and C439SR1 Is a Protein Possessing Novel Thioredoxin-like Activity. *Biochemistry* 31, 9752–9759.

Moore, R. M., Harrison, A. O., McAllister, S. M., and Wommack, K. E. (2018). Iroki: automatic customization and visualization of phylogenetic trees. *bioRxiv*. doi:10.1101/106138.

Nordlund, P., and Eklund, H. (1993). Structure and function of the *Escherichia coli* ribonucleotide reductase protein R2. *J. Mol. Biol.* 232, 123–164.

Ormö Mats (1995). Residues important for radical stability in ribonucleotide reductase from *Escherichia coli*. *J. Biol. Chem.* 270, 6570–6576.

Persson, A. L., Eriksson, M., Katterle, B., Pö, S., Sahlin, M., and Sjö, B.-M. (1997). A New Mechanism-based Radical Intermediate in a Mutant R1 Protein Affecting the Catalytically Essential Glu 441 in *Escherichia coli* Ribonucleotide Reductase. *1The J. Biol. Chem.* 272, 31533–31541.

Price, M. N., Dehal, P. S., and Arkin, A. P. (2010). FastTree 2 – Approximately Maximum-Likelihood Trees for Large Alignments. *PLoS One* 5, e9490. doi:10.1371/journal.pone.0009490.

Rose, H. R., Ghosh, M. K., Maggiolo, A. O., Pollock, C. J., Blaesi, E. J., Hajj, V., et al. (2018). Structural Basis for Superoxide Activation of Flavobacterium johnsoniae Class I Ribonucleotide Reductase and for Radical Initiation by Its Dimanganese Cofactor. *Biochemistry* 57, 2679–2693. doi:10.1021/acs.biochem.8b00247.

Rova, U., Adrait, A., Pötsch, S., Gräslund, A., and Thelander, L. (1999). Evidence by mutagenesis that Tyr(370) of the mouse ribonucleotide reductase R2 protein is the connecting link in the intersubunit radical transfer pathway. *J. Biol. Chem.* 274, 23746–51. doi:10.1074/JBC.274.34.23746.

Shannon, P., Markiel, A., Ozier, O., Baliga, N. S., Wang, J. T., Ramage, D., et al. (2003). Cytoscape: A Software Environment for Integrated Models of Biomolecular Interaction Networks. *Genome Res.* 13, 2498–2504. doi:10.1101/gr.1239303.

Sintchak, M. D., Arjara, G., Kellogg, B. A., Stubbe, J., and Drennan, C. L. (2002). The crystal structure of class II ribonucleotide reductase reveals how an allosterically regulated monomer mimics a dimer. *Nat. Struct. Biol.* 9, 293–300. doi:10.1038/nsb774.

Smoot, M. E., Ono, K., Ruscheinski, J., Wang, P.-L., and Ideker, T. (2011). Cytoscape 2.8: new features for data integration and network visualization. *Bioinformatics* 27, 431–432. doi:10.1093/bioinformatics/btq675.

Uhlin, U., and Eklund, H. (1994). Structure of ribonucleotide reductase protein R1. *Nature* 370, 533–539. doi:10.1038/370533a0.

Uppsten, M., Färnegårdh, M., Jordan, A., Eliasson, R., Eklund, H., and Uhlin, U. (2003). Structure of the Large Subunit of Class Ib Ribonucleotide Reductase from *Salmonella typhimurium* and its Complexes with Allosteric Effectors. *J. Mol. Biol.* 330, 87–97. doi:10.1016/S0022-2836(03)00538-2.
